# Alcohol use is common in older patients with fall-related fractures: a secondary analysis from a prospective cohort study using PEth as an objective assessment of consumption

**DOI:** 10.2340/17453674.2026.46961

**Published:** 2026-10-19

**Authors:** Cecilia ROGMARK, Linnea MALMGREN, Anna HOLMGREN, Åsa MAGNUSSON, Helmi-Sisko PYRHÖNEN

**Affiliations:** 1 Department of Orthopaedics, Skåne University Hospital, Malmö; 2 Faculty of Medicine, Lund University, Lund; 3 Department of Geriatrics, Skåne University Hospital, Malmö; 4 Institute for Clinical Neuroscience, Karolinska Institute, Stockholm, Sweden

## Abstract

**Background and purpose:**

Alcohol increases the risk of falls and complications, but data on alcohol intake among trauma patients is unreliable. Excessive alcohol use can contribute to excess frailty and confusion in frail individuals and increase fall risk in otherwise robust older people. Phosphatidylethanol (PEth) is a biomarker accumulating through repeated alcohol intake, i.e., suitable for screening long-term consumption. Our primary aim was to describe alcohol consumption in older individuals admitted after a fall-related fracture. Our secondary, exploratory aims were to investigate the association between alcohol, frailty, and short-term clinical outcomes in these individuals.

**Methods:**

This prospective observational cohort study included those ≥ 65 years admitted due to acute fall-related fracture February 1, 2023–August 31, 2024. PEth was taken at admission. Predefined norm values classified low/no consumption as < 0.05, intermediate 0.05–0.30 and hazardous consumption > 0.30 *µ*mol/L. Frailty status was assessed by the Clinical Frailty Scale (CFS). Our secondary aim was addressed using multivariable logistic regression on a propensity score-matched cohort.

**Results:**

Of the 505 patients included, 13% had PEth indicating hazardous consumption, 8.9% intermediate consumption, and 78% had low/no consumption. Of the non-frail patients, 18% had hazardous and 11% intermediate consumption. 19% of all women and 29% of all men had continuous alcohol consumption (defined as PEth ≥ 0.05 *µ*mol/L). In the matched cohort, alcohol consumption was associated with a higher odds ratio (OR) of hyponatremia (OR 2.3, 95% confidence interval [CI] 1.1–4.5, P = 0.02). A trend towards lower odds of 90-day mortality (OR 0.2, CI 0.1–1.0, P = 0.05) was seen.

**Conclusion:**

A minimum of 1/5 older patients with fall-related fractures have continuous alcohol consumption. Non-frail patients had a higher intake than frail individuals. Alcohol consumption was associated with higher odds of hyponatremia. Otherwise, no apparent negative effects were identified in the short term.

Although risk factors for fall-related fractures are well known, prevention is difficult [1]. Alcohol as a fall-risk factor is less studied [2-4]. During 2006–2021 in Sweden, the self-reported hazardous alcohol consumption increased in age groups 45–64 and 65–84 years. For women > 65 years, an increase from 3% to 8% was seen, and for men 9% to 14% [5]. A population cohort study described “a dramatic increase in at-risk consumption among 70-year-olds from the 1970s to the mid-2010s … particularly pronounced among women” [6]. In the acute trauma setting, the addition of untreated withdrawal symptoms can put excess stress on both somatic and psychological function. Also, alcohol consumption has been associated with increased risk of postoperative complications, such as wound complications and infections [7]. Still, the readiness to identify and offer treatment for these individuals is low.

In a prospective cohort study of 218 younger adults with hip fractures, the validated AUDIT (Alcohol Use Disorder Identification Test) questionnaire identified hazardous alcohol consumption in 25% of women and 31% of men. A parallel routine clinical evaluation identified 25% of the women and 26% of the men to have alcohol use disorder (AUD). 8% women and 13% men remained undetected by the clinical evaluation, even if they had AUDIT scores indicating hazardous/harmful use [8]. Alcohol consumption might also be underreported in self-administered questionnaires [9,10].

Phosphatidylethanol (PEth), is a direct metabolite formed only in the presence of alcohol, which makes it a highly specific and sensitive biomarker. PEth has been shown to correlate with self-reported drinking and can be used in older individuals as well [11-14]. By integrating PEth into the routine admission blood tests on the orthopedic ward, individuals with harmful consumption can be better identified.

The primary aim of the study was to map the use of alcohol among older individuals hospitalized after a fall-related fracture by using the biomarker PEth. Secondarily, as an exploratory aim, we investigated the association between alcohol, frailty, and short-term clinical outcomes in these patients.

## Methods

### Study design and setting

This study is reported according to STROBE guidelines.

In this prospective cohort study, all patients aged 65 or older with fall-related injuries, admitted to the Department of Orthopedics at Skåne University Hospital in Malmö, Sweden, during February 1, 2023–August 31, 2024, were assessed for eligibility (n = 1,569). This is a level 1 trauma center with a catchment population of 400,000.

According to a newly introduced protocol at the department, PEth was included in an updated admission blood test panel, taken on the day after admission. All patients 65 years and older admitted due to fall-related fracture were supposed to have these blood tests taken, including PEth. Data on blood tests, baseline characteristics, and outcome measures was collected from medical journals continuously during the inclusion period and entered into a RedCap (https://project-redcap.org/) database. Individuals were followed up for 30 days regarding complications and 90 days regarding death.

### Participants

Of the 1,569 patients aged 65 or older with fall-related injuries, individuals were excluded according to the following exclusion criteria: admission via route other than the Emergency Department (n = 204), fall > 7 days ago (n = 93), not an actual fall (n = 94), resident in other region (n = 45), no fracture (n = 35), and patient choosing to opt out (n = 4). 1,094 patients were eligible for inclusion, of whom 589 individuals were excluded due to missing PEth, resulting in inclusion of 505 individuals. As the inclusion period was predefined, no formal sample size calculation regarding study size was made.

### PEth and alcohol consumption

To detect PEth in blood, more than 1 week of regular consumption of more than moderate amounts of alcohol is needed, and a single dose gives only unmeasurable PEth levels [12,15,16]. PEth has a biphasic half-life that can vary from 4 to 10 days, and elevated values can be detected up to several weeks after alcohol intake, depending on whether it was a single or repeated intake [12,15,16].

A consensus statement defined these thresholds: < 20 ng/mL—compatible with abstinence or low alcohol consumption; ≥ 20 ng/mL but <200 ng/mL—alcohol consumption; ≥ 200 ng/mL—strongly suggestive of chronic excessive alcohol consumption. “Chronic excessive alcohol consumption” is in turn said to correspond to an average consumption of pure ethanol on a single drinking day over a prolonged duration of ≥ 60 g for men and ≥ 40 g for women [15].

### Variables

The PEth thresholds used in our laboratory were < 0.05 *µ*mol/L as no or low consumption, 0.05–0.29 *µ*mol/L as moderate consumption and ≥ 0.3 *µ*mol/L as harmful consumption [13]. PEth values ≥ 0.05 *µ*mol/L are in this study termed “continuous alcohol consumption.”

Frailty status was evaluated using the Clinical Frailty Score [17] as part of routine care when arriving on the ward. The assessment was conducted by occupational therapists, with assistance from ward physicians and/or nurses when needed. For analysis, CFS scores were dichotomized into non-frail (CFS 1–4) or frail (CFS 5–9). As 101 patients with PEth lacked frailty assessment, these individuals were categorized based on their type of living: living in own home was deemed non-frail, and institutional living as frail. 256 patients were non-frail and 249 frail.

The outcome variables studied in the secondary analyses were extracted from the medical records and were defined as:

- 90-day and 30-day mortality, retrieved from medical records and cross-checked against Swedish national population register.
- Confusion within 30 days—acute mental disorientation documented at least twice, either as a new onset or aggravation of pre-existing cognitive impairment. No formal assessment was undertaken.
- Infections within 30 days—pneumonia, culture-verified urinary tract infections, sepsis, soft tissue infections, or surgical site infections, diagnosed by ward physicians in collaboration with internal and infectious medicine specialists.
- Surgical complications within 30 days—wound leakage, superficial and deep surgical site infections, implant failure, dislocation, or other fracture/surgery-related complications.
- Pressure sores within 30 days—known pre-existing pressure sores were excluded.
- Low albumin, measured on admission—normal reference interval of the laboratory for albumin was 36–45 g/L if age 41–70 years and 34–45 g/L if age > 70 years. Values under the reference intervals defined hypoalbuminemia.
- Handgrip strength—measured with the Jamar tool on day 3–4 as inpatient or post-surgery, best out of 3; sarcopenia was defined as < 16 kg for women, < 27 kg for men [18].
- Hyponatremia measured on admission — < 135 mmol/L.

### Statistics

The primary aim was addressed descriptively. Participants were grouped by alcohol consumption level based on PEth levels, and the distributions of age, sex, frailty, and cause of injury across these groups are presented. Categorical data is presented as proportions, while continuous data is reported as means (with standard deviations [SD]) or medians (with interquartile ranges [IQR]), depending on the data distribution.

To address the secondary aim, propensity score matching (PSM) with a 1:3 nearest neighbor matching approach and a caliper of 0.2 was conducted to balance baseline characteristics between individuals with continuous alcohol consumption (defined as PEth ≥ 0.05 μmol/L) and low consumption (PEth < 0.05 μmol/L). Separate analyses were performed for (i) the entire cohort, and (ii) for women and men separately. Propensity scores were estimated using logistic regression with age (continuous) and frailty (non-frail or frail) as covariates. For the entire cohort, sex was included as an additional covariate.

For the balance diagnostics after matching, the following thresholds were predefined as satisfactory: standardized mean difference (SMD) < 10%, Rubin’s B < 25% and Rubin’s R between 0.5 and 2.0 (Supplementary Tables S1–S3).

Logistic regression, adjusted for the matching variables, was performed for each outcome in the propensity score-matched cohorts using Stata’s logistic command.

All analyses were performed using Stata version 18.0 (StataCorp, College Station, TX, USA), and a 2-sided P value <0.05 was considered statistically significant.

### Missing data and bias

Individuals missing PEth measurements were excluded from main analyses as presented earlier. Table 1 presents baseline characteristics of those with vs without PEth measurement. For the matched analyses, individuals who missed albumin and handgrip strength were excluded from the regression analyses. Because baseline characteristics between those with no/low vs continuous alcohol consumption differed remarkably, we performed propensity score matching by age, sex, and frailty (as already described) and further adjusted the outcome models for these matching variables to minimize residual confounding for the secondary analyses.

**Table 1:** Patient charactistics. “PEth available”: PEth was taken as part of admission blood tests. “No PEth”: no PEth was analysed. “All eligible”: all eligible patients with fall-related fractures. Values are count (%) or as specified

Factor	PEth available n = 505	No Peth n = 589	All eligible n = 1,094
Women	345 (68)	409 (69)	754 (69)
Age, mean (SD)	82 (8.4)	83 (7.9)	83 (8.1)
Body mass index, median (IQR)	24 (21–27)	24 (21–27)	24 (21–27)
Clinical Frailty Scale score		
1–4	182 (36)	152 (26)	334 (31)
5–9	222 (44)	215 (37)	437 (40)
Missing	101 (20)	222 (38)	323 (30)
Living alone	274 (54)	296 (50)	570 (52)
Information in medical records on alcohol		
Current risk consumption	46 (9.1)	25 (4.2)	71 (6.5)
Previous risk consumption	22 (4.4)	29 (4.9)	51 (4.7)
Moderate or no consumption	279 (55)	342 (58)	621 (57)
Missing information	158 (31)	193 (33)	351 (32)
Smoking		
Active smoker	72 (14)	54 (9.2)	126 (12)
Ex-smoker	120 (24)	159 (27)	279 (26)
Never-smoker	230 (46)	283 (48)	513 (47)
Missing information	83 (16)	93 (16)	176 (16)
Fall reason		
Dizziness	37 (7.3)	44 (7.5)	81 (7.4)
Infection	7 (1.4)	5 (0.9)	12 (1.1)
Seizure	0	1 (0.2)	1 (0.1)
Cardiovascular/arrythmia	6 (1.2)	5 (0.9)	11 (1.0)
Intoxication	22 (4.4)	15 (2.6)	37 (3.4)
Other reason	440 (87)	522 (89)	962 (88)
Type of fracture		
Hip fracture	334 (66)	310 (53)	644 (59)
Other fractures, listed below ^**a**^	171 (34)	279 (47)	450 (41)
- Femoral shaft or distal femoral	25	24	49
- Ankle	35	39	74
- Pelvic	26	43	69
- Other fracture of the lower extremity	21	39	60
- Proximal humerus fracture	36	43	79
- Distal radius fracture	14	16	30
- Other fracture in the upper extremity	31	48	79
- Spinal fractures	8	59	67
- Other than mentioned above	3	5	8

a 54 patients had 2 simultaneous fractures.

### Ethics, registration, data sharing plan, funding, and disclosures

The project is approved by the Swedish Ethics Review Board (2022-01997-01). Given the standardized model of care, no active consent was deemed necessary. Patients were given written information, and an opt-out model was used. The study protocol was registered at ClinicalTrials.gov (NCT05712252).

Data may be shared upon reasonable request. The project was supported by grants from the Greta and Johan Kock Foundation, H. Järnhardt Foundation, Skåne University Hospital Research Fund, the Research and Development Council of Region Skåne, and the Swedish Research Council funding for clinical research in medicine. None of the funders had influence on the scientific work of this study. CR has received lecture fees from Swemac and LINK, otherwise none of the authors have any financial or competing interests related to the subject. Completed disclosure forms for this article following the ICMJE template are available on the article home page, doi: 10.2340/17453674.2026.46961

## Results

PEth was measured in 505 patients, while 589 patients were excluded from further analyses due to missing PEth measurement (Figure). The mean age (82 years, SD 8.4 vs 83, SD 7.9) and the proportion of women (68% vs 69%) were comparable between patients with measured PEth and those missing PEth measurement. The group with PEth comprised more patients with hip fractures, 66% vs 53%. Information in medical records on past or current alcohol risk consumption was also somewhat more common among those who had PEth taken (14% vs 9%). Unexplained, simple falls caused the injury in 87% and 89% (see Table 1).

**Figure:**
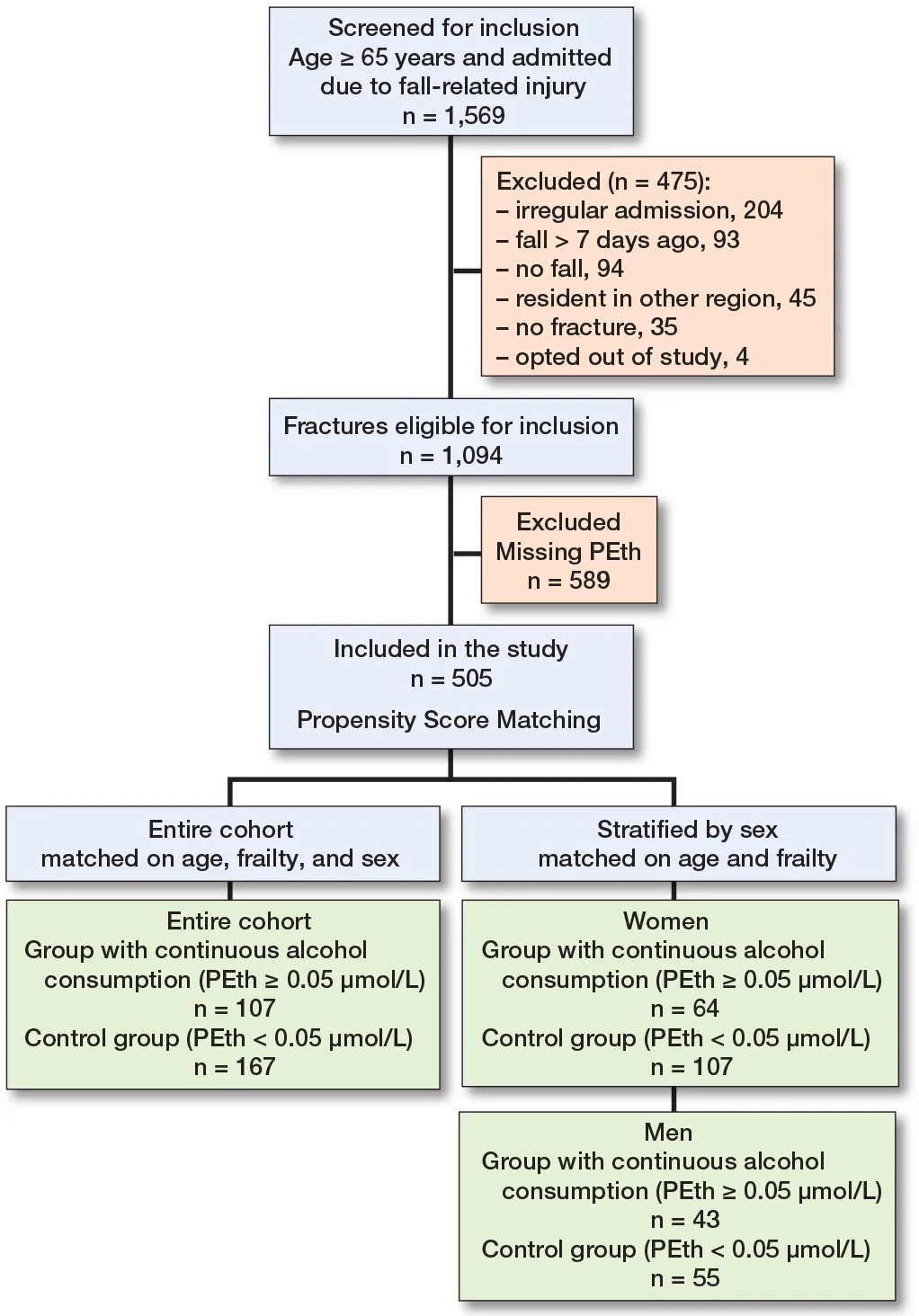
Flowchart of screened, excluded, and included patients. PEth: Phosphatidylethanol.

### Primary analysis: alcohol consumption

66 of 505 participants (13%) had PEth indicating hazardous consumption, 45 (8.9%) had intermediate consumption, while 394 (78%) had low/no consumption. Of the 256 non-frail patients, corresponding percentages were 18% hazardous, 11% intermediate, and 71% low/no consumption. PEth indicated hazardous consumption in 8% of those assessed as frail.

64 women and 47 men had continuous alcohol consumption, defined as PEth ≥ 0.05 μmol/L (19% of all women, 29% of all men). Individuals with continuous alcohol consumption were younger than those with low/no consumption (77 vs 84 years). 24% of non-frail women (38/161) and 38% of non-frail men (36/95) had continuous alcohol consumption. In the group with continuous alcohol consumption, as many as 31% had notes in their medical records stating that they reported “moderate or no consumption”; 19% of them had intoxication as cause of injury, compared with 0.3% in the group with low or no consumption (Table 2).

**Table 2:** Patient characteristics for groups dichotomized on alcohol consumption. Values are count (%) or as specified

Factor	Alcohol consumption		All patients with PEth
	low or no ^a^	continuous ^b^	
Total	394 (78)	111 (22)	505
Women	281 (71)	64 (58)	345 (68)
Age, mean (SD)	84 (7.0)	77 (8.1)	82 (8.4)
Clinical Frailty Scale score			
1–4	118 (30)	64 (58)	182 (36)
5–9	185 (47)	37 (33)	222 (44)
Missing	91 (23)	10 (9.0)	101 (20)
Information in medical records on alcohol			
Current risk consumption	2 (0.5)	44 (40)	46 (9.1)
Previous risk consumption	11 (2.8)	11 (9.9)	22 (4.4)
Moderate or no consumption	245 (62)	34 (31)	279 (55)
Missing information	136 (34)	22 (20)	158 (31)
Smoking			
Active smoker	32 (8.1)	40 (36)	72 (14)
Ex-smoker	91 (23)	29 (26)	120 (24)
Never-smoker	201 (51)	29 (26)	230 (46)
Missing information	80 (18)	13 (12)	83 (16)
Fall reason			
Dizziness	29 (7.4)	8 (7.2)	37 (7.3)
Infection	7 (1.8)	0	7 (1.4)
Seizure	0	0	0
Cardiovascular/arrythmia	5 (1.2)	1 (0.9)	6 (1.2)
Intoxication	1 (0.3)	21 (18.9)	22 (4.4)
Other reason	356 (90)	84 (76)	440 (87)

a PEth < 0.05 *µ*mol/L;

b PEth ≥ 0.05 *µ*mol/L

### Secondary analysis: association between alcohol and clinical outcome

After matching, covariate balance improved markedly. SMDs were below 10% for all covariates except for frailty in the female cohort (SMD 10.5%), a minor residual imbalance that was considered acceptable in the light of overall diagnostics. Rubin’s B and Rubin’s R were within the predefined ranges across the matched cohorts (see Supplementary Tables S1–S3).

#### Entire matched cohort

111 individuals had continuous alcohol consumption. The 394 individuals with PEth < 0.05 μmol/L served as a control group. After matching on age, frailty and sex, 107 individuals were compared with 167 controls. In logistic regression adjusted for age, frailty, and sex, alcohol consumption was associated with higher odds of hyponatremia (odds ratio [OR] 2.3, 95% confidence interval [CI] 1.1–4.5, P = 0.02), a trend towards lower odds of 90-day mortality (OR 0.2, CI 0.1–1.0, P = 0.05), and higher odds of hypoalbuminemia (OR 1.7, CI 1.0–2.8, P = 0.06). No association was shown for other outcomes (Table 3).

**Table 3:** Logistic regression showing association between continuous alcohol consumption and each outcome in the entire propensity score-matched cohort, adjusted for age, sex, and frailty. 107 individuals with continuous alcohol consumption were compared with 167 controls. Continuous alcohol consumption defined as PEth ≥ 0.05 μmol/L, low/no consumption (PEth < 0.05 μmol/L) used as reference. Propensity scores were estimated using logistic regression with age (continuous), sex and frailty as covariates

Factor	Odds ratio (CI)	P value	Event rate
90-day mortality	0.2 (0.0–1.0)	0.05	17/274
30-day mortality	0.2 (0.0–1.6)	0.1	9/274
Confusion	0.7 (0.4–1.4)	0.4	59/274
Infection	0.7 (0.3–1.6)	0.4	31/274
Surgical complication	0.8 (0.4–1.8)	0.6	31/274
Pressure sore	0.6 (0.2–1.6)	0.3	19/274
Hypoalbuminemia	1.7 (1.0–2.8)	0.06	131/251
Handgrip strength	1.0 (0.5–2.0)	0.9	81/171
Hyponatremia	2.3 (1.1–4.5)	0.02	41/274

CI: 95% confidence interval. Event rate: total number of events/total number analyzed.

#### Female cohort

After matching on age and frailty, 64 women with continuous alcohol consumption were compared with 106 female controls. In logistic regression adjusted for age and frailty, alcohol consumption was not associated with any outcome, besides a trend towards higher odds of hyponatremia (OR 2.2, CI 1.0–5.0, P = 0.054) (Table 4).

**Table 4:** Logistic regression showing association between continuous alcohol consumption and each outcome in the female propensity score-matched cohort, adjusted for age and frailty. 64 women with continuous alcohol consumption were compared with 106 female controls

Factor	Odds ratio (CI)	P value	Event rate
90-day mortality	0.3 (0.0–3.1)	0.3	7/170
30-day mortality	Not applicable, too few observations		3/170
Confusion	1.0 (0.4–2.1)	0.9	37/170
Infection	1.0 (0.3–2.8)	0.9	17/170
Surgical complication	0.7 (0.3–2.0)	0.5	19/170
Pressure sore	0.9 (0.2–3.6)	0.8	9/170
Hypoalbuminemia	1.2 (0.6–2.3)	0.6	83/157
Handgrip strength	0.9 (0.4–1.9)	0.7	50/114
Hyponatremia	2.2 (1.0–5.0)	0.05	32/170

For definitions, see Table 3.

#### Male cohort

After matching, 43 men with continuous alcohol consumption were compared with 55 men in the control group. In logistic regression adjusted for age and frailty, alcohol consumption was not associated with any outcome, besides a trend towards higher odds of hypoalbuminemia (OR 2.5, CI 1.0–6.3, P = 0.06) (Table 5).

**Table 5:** Logistic regression showing association between continuous alcohol consumption and each outcome in the male propensity score-matched cohort, adjusted for age and frailty. 43 men with continuous alcohol consumption were compared with 55 men in the control group

Factor	Odds ratio (CI)	P value	Event rate
90-day mortality	0.2 (0.0–2.0)	0.2	7/98
30-day mortality	Not applicable, too few observations		3/98
Confusion	0.4 (0.1–1.3)	0.1	21/98
Infection	0.8 (0.2–3.4)	0.7	9/98
Surgical complication	1.0 (0.3–3.8)	0.9	11/98
Pressure sore	0.4 (0.1–2.2)	0.3	8/98
Hypoalbuminemia	2.5 (1.0–6.3)	0.06	49/88
Handgrip strength	1.6 (0.5–4.7)	0.4	30/64
Hyponatremia	2.1 (0.4–10.0)	0.3	9/98

For definitions, see Table 3.

## Discussion

Our primary aim was to describe alcohol consumption in older individuals admitted after a fall-related fracture. Our secondary, exploratory aims were to investigate the association between alcohol, frailty, and short-term clinical outcomes in these individuals. We found alcohol use to be relatively common among people aged 65 years or older admitted due to fall injuries, particularly among the non-frail. Based on the biomarker PEth, 13% had hazardous alcohol intake, and another 9% had intermediate elevation indicating continuous intake. This biomarker-based consumption rate is in line with a national population sample where 8% women and 13% men aged 65–84 years reported hazardous alcohol consumption [5].

Our cohort comprised different people aged 65 years and older. Some were still of working age whilst other were more or less frail, and a few moribund. We found that patients considered frail less often used alcohol, presumably having other causes of injury than non-frail individuals. In the final stage of life, alcohol drinking might no longer be wished for. In the aforementioned population sample, only 2% of both sexes > 84 years reported hazardous alcohol consumption [5]. When depending on help from others, it can also be practically difficult to get hold of alcohol; nevertheless 1 in 12 of our frail patients had hazardous consumption.

When caring for non-frail patients with fall-related trauma, physicians should be aware that a quarter of the women and more than one-third of the men have continuous alcohol intake. Post-trauma care gives an opportunity to address alcohol as a cause of injury as well as any concerns from the patient regarding his/her drinking pattern. This may be especially relevant in this group of older adults, where polypharmacy—including high-risk medications such as anticoagulants—is prevalent.

Generally, increasing focus is put on alcohol consumption among older individuals. “Moderate consumption” of alcohol has gained positive attention, due to the association with better quality of-life [9], better bone health [18,19], and less frailty [20]. Some types of alcohol use can be a marker of a positive and active lifestyle [21]. These notions may contribute to the increased alcohol consumption among old people observed in many countries [22]. On the other hand, an aging body will tolerate lower amounts of alcohol [23], and the maintenance of working-age alcohol habits into retirement age may lead to health issues [24]. Women risk injuries at a relatively low alcohol intake compared with men [25]. To try to mitigate the alcohol intake in those treated due to an acute fracture can be challenging [26] but is important as alcohol may delay fracture healing and increases the risk of infection [27,28].

We found higher odds of hyponatremia and a trend towards hypoalbuminemia among those with continuous alcohol consumption. Hyponatremia is a well-known clinical problem in patients with high alcohol intake [29]. Low albumin, as a surrogate marker of poor nutritional status and chronic inflammation, or liver disease, has been associated with higher mortality and postoperative complications in hip fracture patients [30]. Interestingly, we found no negative effect of alcohol consumption on other outcome measures. Quite the contrary, it was associated with a trend toward lower mortality at 90 days.

The “capacity to drink” may also be associated with better health [20,31], whilst apparent negative effects of high alcohol intake will coincide with a point in time where the individual is no longer capable of drinking.

PEth has a minimal risk of false positive findings and a higher sensitivity than other alcohol markers such as carbohydrate deficient transferrin (CDT), mean corpuscular volume and gamma-GT [12,16]. There is a significant correlation between the PEth value and the amount of alcohol consumed, but the individual response to PEth varies. A meta-analysis identified body mass index and hemoglobin amongst other biological factors to interfere with the sensitivity, whereas age and sex seem to play little or no role [11]. A measurable PEth confirms alcohol intake, but the amount and time of intake cannot be determined with certainty. The half-life of PEth also varies and has been stated in studies to be between 4 and 10 days. PEth can therefore be said to reflect a couple of weeks’ to the last month’s alcohol intake [12,15,16].

PEth is suggested as a screening method for alcohol use and studies report the biomarker’s usefulness when caring for patients with withdrawal symptoms, when planning liver transplantation, and in emergency departments [32-34]. In a Brazil cohort of younger trauma patients, “any level of alcohol misuse” based on AUDIT-C questionnaires was found in 64 of 238 participants (27%). PEth was useful to identify 12 additional participants who reported their alcohol use in the last year to be nil (AUDIT-C = 0) [10]. PEth correlates better to self-reported intake than other biomarkers, while it also appears to be a more reliable assessment of alcohol consumption than self-reported intake [14].

### Limitations

PEth values were available for just under half of included patients. Despite frequent reminders, prepacked test tubes, and lab forms, nurses often failed to take the tests. This was explained by either heavy workload or external personnel on rotation not familiar with the new routines. When comparing patients with and without a PEth measurement, the differences in age and sex were small. There was a tendency for more documented alcohol use in those who had PEth taken. On the other hand, one-third of all patients did not have any notes at all on alcohol in their medical records. There were more patients with hip fractures in the group with PEth taken, most likely reflecting better adherence among nurses to taking blood tests as part of a preoperative hip fracture work-up. Based on the clinical observations during the study period, we consider most of the missing PEth values to be missing at random.

Another limitation is that the study primarily reports short-term outcomes. Medical complications were chosen to be assessed only within 30 days, to have a clear connection with the initial injury. Further, the sample sizes, particularly in the matched analyses, were rather small, increasing statistical uncertainty. Finally, due to a high rate of chronic and acute cognitive impairment, we have chosen not to complete the assessment with self-administered questionnaires for this cohort.

### Strengths

The use of the sensitive and specific biomarker PEth enabled inclusion of even the frailest patients with cognitive impairment, where self-reported alcohol use may not be applicable. PEth is an established method for screening of alcohol use [33,34]. Through an opt-out consent procedure approved by the ethics committee, the risk of selection bias is considered very small. Mortality is a highly reliable outcome, based on linkage through the unique personal identity number used in our country. The medical complications could be identified with high completeness, as long as they occurred during initial hospital stay or led to a new hospital contact within 30 days.

### Clinical perspective

The rationale for introducing routine PEth testing on admission after fall injury was that it is already recommended and used in our region as part of the assessment when evaluating cognitive disease. Now, after not seeing any association with negative short-term outcome besides hyponatremia, it does not seem meaningful to screen all orthogeriatric patients. On the other hand, we think our results support PEth as a useful biomarker as part of an individual assessment, when alcohol is suspected as a contributing factor to the injury. We noted a certain underreporting of excess alcohol use in the medical records. Objective measurement of alcohol consumption is also clinically important to prevent withdrawal syndrome and cognitive deterioration in the acute setting.

Because over 1 in 5 individuals hospitalized due to fall injuries used alcohol continuously, addictive medicine competence on the orthogeriatric ward may be relevant. A specialized nurse/therapist may have better chances of reaching an individual with harmful consumption if the treatment sessions are part of the regular orthogeriatric care pathway, in contrast to a more stigmatizing referral to the department of addictive medicine.

### Conclusion

More than 1 in 5 individuals hospitalized due to fall injuries had continuous alcohol consumption. Stratifying by frailty, close to one-third of non-frail patients had continuous consumption, which is higher than the background population. Frail individuals were less likely to have continuous alcohol consumption. Alcohol consumption was associated with higher odds of hyponatremia. The association with lower 90-day mortality was on the verge of significance.

## Supplementary Material



## Supplementary data

Supplementary Tables S1–S3 are available as supplementary data on the article page, doi: 10.2340/17453674.2026.46961

## Notes

CR, HSP, LM, and ÅM designed and conducted the main cohort study. CR, HSP, and AH collected and organized data. HSP did the statisti¬cal analyses. CR wrote the manuscript draft. All authors participated in revision of the manuscript and approved the submitted version of the manuscript.

The authors would like to thank the FFF research team for their valuable contributions and the research administrator Marika Bergman for her essential support throughout the study.

Handling co-editors: Per Henrik Randsborg and Philippe Wagner

*Acta* thanks Bjarke Viberg for help with peer review of this manuscript.
