# Supplementary material for: Alcohol use is common in older patients with fall-related fractures: a secondary analysis from a prospective cohort study using PEth as an objective assessment of consumption
Source: Acta Orthop. 2026 Oct 19;97:743–50. doi: 10.2340/17453674.2026.46961 (PMC13678457; doi:10.2340/17453674.2026.46961)
Supplement: Supplementary file 1 [file ActaO-97-46961-s1.pdf]

Supplementary Data

Supplementary Table S1. A) Balance diagnostics and B) Rubin's B and R diagnostics for the entire cohort, pre- and post-matching results are shown.

**A)**	**Before matching**				**After matching**				**SMD (%bias)**	**Variance ratio (Treated/Control)**
**Variable**	PEth ≥0.05 (Treated) n = 111	Control n = 394	t	P value (P>|t|)	PEth ≥0.05 (Treated) n = 107	Control n = 167	t	P value (P>|t|)	Before → After	After
**Age**, mean	77.3	83.7	–7.49	<0.001	77.7	77.9	–0.16	0.876	–79.8 → -2.1	1
**% Female**	57.7	71.3	2.75	0.006	59.8	62.3	0.37	0.71	28.8 → 5.2	1.02
**% Frail**	33.3	53.8	–3.86	<0.001	34.6	29.9	0.73	0.467	–42.1 → 9.6	

**B) Sample**	**Pseudo-R2**	**LR chi^2**	**P >chi^2**	**Mean absolute SMD (%bias/SMD)**	**Median absolute SMD**	**B**	**R**	**%Var**
Unmatched	0.111	58.87	0	50.2	42.1	85.0 [a]	1.08	0
Matched	0.003	0.9	0.83	5.6	5.2	12.9	1.07	0

[a] if B>25%, R outside [0.5; 2]

SMD = standardized mean difference.

Supplementary Table S2. A) Balance diagnostics and B) Rubin's B and R diagnostics for the female cohort, pre- and post-matching results are shown. SMD= standardized mean difference.

A)	Before matching				After matching				SMD (%bias)	Variance ratio (Treated/Control)
**Variable**	PEth ≥0.05 (Treated) n = 64	Control n = 281	t	P value (P>|t|)	PEth ≥0.05 (Treated) n = 64	Control n = 106	t	P value (P>|t|)	Before → After	After
**Age,** mean	78	84.3	–5.72	<0.001	78	78.2	–0.16	0.876	–80.4 → -2.7	1
**% Frail**	40.6	56.2	–2.26	0.024	40.6	35.4	0.6	0.548	–31.5 → -10.5	.

B) Sample	Pseudo-R2	LR chi^2	P >chi^2	Mean absolute SMD (%bias/SMD)	Median absolute SMD	B	R	%Var
Unmatched	0.096	31.83	0	55.9	55.9	81.4 [a]	0.89	0
Matched	0.003	0.45	0.8	6.6	6.6	11.7	1.04	0

[a] if B>25%, R outside [0.5; 2]

Supplementary Table S3. A) Balance diagnostics and B) Rubin's B and R diagnostics for the male cohort, pre- and post-matching results are shown. SMD= standardized mean difference.

**A)**	**Before matching**				**After matching**				**SMD (%bias)**	**Variance ratio (Treated/Control)**
**Variable**	PEth ≥0.05 (Treated) n = 47	Control n = 113	t	P value (P>|t|)	PEth ≥0.05 (Treated) n = 43	Control n = 55	t	P value (P>|t|)	Before → After	After
**Age,** mean	76.3	82.3	–4.3	<0.001	77.3	77.3	0.04	0.966	–72.4 → 0.9	0.98
**% Frail**	23.4	47.8	–2.92	0.004	25.6	26.4	–0.08	0.936	–52.3 → –1.7	

**B) Sample**	**Pseudo-R2**	**LR chi²**	**P >chi²**	**Mean absolute SMD (%bias/SMD)**	**Median absolute SMD**	**B**	**R**	**%Var**
Unmatched	0.11	21.27	0	62.3	62.3	81.9 [a]	1.36	0
Matched	0	0.01	0.99	1.3	1.3	2.3	0.97	0

[a] if B>25%, R outside [0.5; 2]
